# Keeping pace with the healthcare transformation: a literature review and research agenda for a new decade of health information systems research

**DOI:** 10.1007/s12525-021-00484-1

**Published:** 2021-07-17

**Authors:** Nadine Ostern, Guido Perscheid, Caroline Reelitz, Jürgen Moormann

**Affiliations:** 1 Chair for Digitalization and Process Management, Philipps-University Marburg, Universitätsstraße 24, 35037 Marburg, Germany; 2 Frankfurt School of Finance & Management, ProcessLab, Adickesallee 32-34, 60322 Frankfurt am Main, Germany

**Keywords:** Healthcare, Health information systems research, Research agenda, I0, I1

## Abstract

**Background:**

Accelerated by the coronavirus disease 2019 (Covid-19) pandemic, major and lasting changes are occuring in healthcare structures, impacting people's experiences and value creation in all aspects of their lives. Information systems (IS) research can support analysing and anticipating resulting effects.

**Aim:**

The purpose of this study is to examine in what areas health information systems (HIS) researchers can assess changes in healthcare structures and, thus, be prepared to shape future developments.

**Method:**

A hermeneutic framework is applied to conduct a literature review and to identify the contributions that IS research makes in analysing and advancing the healthcare industry.

**Results:**

We draw an complexity theory by borrowing the concept of 'zooming-in and out', which provides us with a overview of the current, broad body of research in the HIS field. As a result of analysing almost 500 papers, we discovered various shortcomings of current HIS research.

**Contribution:**

We derive future pathways and develop a research agenda that realigns IS research with the transformation of the healthcare industry already under way.

**Supplementary Information:**

The online version contains supplementary material available at 10.1007/s12525-021-00484-1.

## Introduction

Particularly since the last decade, IT has opened up new opportunities for ‘ehealth’ through telemedicine and remote patient monitoring, alongside potential improvements in the cost-effectiveness and accessibility of health care (Chiasson & Davidson, 2004). Accordingly, health information systems (HIS) research has come to focus on how healthcare organizations invest in and then assimilate HIS, looking in particular at the impact of digitalization on healthcare costs, healthcare quality, and patient privacy (Chen et al., 2019; Park, 2016).

Less attention has been paid to issues such as mobile health, health information interchange, digital health communities, and services that change customer expectations and may lead to major disruptions (Chen et al., 2019; Park, 2016). These topics, however, are becoming increasingly important due to the penetration of the user and health market by external players, especially tech companies, providing services such as fitness trackers, and surveillance software for patient monitoring in hospitals (Gantori et al., 2020). Modern IT, thus, becomes a catalyst to provide greater operational efficiency, offering new possibilities for tech companies to build new health-centred business models and services (Park, 2016).

The ways in which tech companies are entering the healthcare industry can be seen amid the spread of coronavirus disease 2019 (Covid-19), which is pushing healthcare systems to the edge of their capacities (Worldbank, 2020). In this extraordinary condition, the pandemic has provided an additional opportunity for tech companies that were hitherto not active or not allowed to enter the healthcare industry (Gantori et al., 2020).

We are currently seeing how entering the healthcare market is actually taking place, particularly in the USA, where tech companies are increasingly offering services to help address some of the problems associated with Covid-19. Google’s subsidiary Verily, for instance, facilitates the automation of coronavirus symptom screening and provides actionable, up-to-date information that supports community-based decision-making (Landi, 2020). Although the collaboration with Verily assists the US government in tracking cases to identify the spread of the virus, it is reasonable to suggest that Verily probably did not launch the screening tool out of altruism. In fact, to receive preliminary screening results via the Verily app, citizens have to log into their personal Google account (Greenwood, 2020). This allows Verily to gain immense value by aggregating huge, structured data sets and analyse them to come up with new health services, such as better tools for disease detection, new data infrastructures, and insurance offerings that – for better or for worse – may outplay current healthcare providers and even disrupt whole healthcare ecosystems (CB Insights, 2018). Similarly, Amazon has started to provide cloud space through Amazon Web Services to store health surveillance data for the Australian government’s tracing app (Tillett, 2020), and Amazon Care, a division initially responsible for handling internal staff care needs, now cooperates with the Bill and Melinda Gates Foundation to distribute Covid-19 testing kits to US residents (Lee & Nilsson, 2020).

Looking at information systems (IS) researchers’ previous assessments of state-of-the-art healthcare-related IS literature reveals that most scholars seem to have little or no concern for the beginning of those potentially long-lasting changes that are occurring in the healthcare industry (Chen et al., 2019). This is worrying, considering that it is already apparent that the years ahead will be marked by economic volatility and social upheaval as well as direct and indirect health consequences, including sweeping transformations in many of the world’s healthcare systems.

While it is clear that recent developments and the push of tech and platform companies into the healthcare sector can significantly improve the quality of life for billions of people around the world, it will be accompanied by serious challenges for healthcare industries, governments, and individuals (Park, 2016). Technological advances are, for instance, giving rise to a plethora of smart, connected products and services, combining sensors, software, data, analytics, and connectivity in all kinds of ways, which in turns leads to a restructuring of health industry boundaries and the empowerment of novel actors, especially tech and platform companies such as IBM, Google, and Amazon (Park, 2016).

Observing those changes, we need to develop a general understanding of long-term trends such as digitalization and blurring industry boundaries. As the pandemic is only an amplifier of longer-lasting trends, it is likely that the consequences and exogenous effects on the healthcare industry will go far beyond the time of the current pandemic. Given these observations, we wonder whether the IS research domain is ready to capture, understand, and accompany these developments, which require a holistic view of the healthcare industry, its structures, and the interdependencies between incumbents and new entrants. Thus, we argue that it is now time to develop a more comprehensive understanding of these developments and to determine the role that IS research can play by asking: *How can we prepare HIS research to capture and anticipate current developments in the healthcare industry?*

To find answers to this question, our paper provides a literature overview of HIS research by ‘zooming in and zooming out’ (Gaskin et al., 2014) and by drawing on complexity theory (Benbya et al., 2020). Since a healthcare system, like the industry as a whole, can be understood as a complex, digital socio-technical system (Kernick & Mitchell, 2009; Therrien et al., 2017), zooming in and zooming out is a way to view, capture, and theorize the causes, dynamics, and consequences of a system’s complexity. Complex systems are characterized by adaptiveness, openness (Cilliers, 2001), and the diversity of actors and their mutual dependency in the system, meaning that outcomes and research span various levels within these systems, although the boundaries of socio-technical systems are elusive. Assuming that HIS research is just as complex as the socio-technical system investigated, we first zoom in, focusing on concrete research outcomes across levels (i.e., what we can actually observe). Zooming in is followed by zooming out, which means abstracting from the concrete level and embracing the strengths and disparities of overall HIS research on a higher level in which concrete research outcomes are embedded (Benbya et al., 2020). Using this approach, we can capture and understand the complexity of HIS research without losing sight of concrete research issues and topics that drive research in this field.

To do this, we chose a hermeneutic framework to guide us in a thorough review and interpretation of HIS literature and lead us to the following overarching observations: (i) The literature review determines the unique contribution that IS research plays in analysing and advancing the healthcare industry. However, it also shows that we are hardly prepared to take up current developments and anticipate their consequences. (ii) The reason for this unpreparedness is that we currently neglect the ecosystem perspective and thus ignore holistic approaches to resolve the striking number of interrelated issues in HIS research. (iii) Based on the unique insights of this literature review, our paper provides a research agenda in which we use complexity theory to discuss the consequences of current developments. This theory assists IS researchers not only to better understand developments and implications thereof for the healthcare industry (and thus HIS research) but also to create a meaningful impact on the future of this industry. Since we have limited our research explicitly to the IS domain, our results may not be generally applicable to other healthcare research domains and we do not claim to provide an overview of the literature in the field of HIS research. However, while IS researchers cannot solve the pandemic directly, preparing them by providing a new research agenda will support them in developing concepts and applications, thereby helping them to overcome the negative effects of the pandemic. In our opinion, it is particularly important that IS research, and especially HIS-related research, obtains a deeper understanding of the needed transformation that is caused by digitalization and the emergence of new players catalysed by the current pandemic.

The remainder of this paper is structured as follows. The next section is concerned with the hermeneutic framework used to conduct the systematic literature review. After explaining the hermeneutic approach and the research steps, we elaborate on the key findings by zooming in; that is, we focus on the key results that emerge from analysing and interpreting the literature for each of the phases defined in the course of the literature sorting process. We then concentrate on zooming out, emphasizing the patterns and interdependencies across phases, which helps us determine the state of HIS research. The results of both parts of the literature review – i.e., zooming in and zooming out (Benbya et al., 2020; Gaskin et al., 2014) – support us in identifying strengths, as well as drawbacks, in HIS research. On this basis, we develop a research agenda that provides future directions for how HIS research can evolve to anticipate the impending transformation of the healthcare industry.

## Literature review: a hermeneutic approach

To answer our research question, we conducted a literature review based on hermeneutic understanding. In particular, we followed Boell and Cecez-Kecmanovic (2014). They proposed a hermeneutic philosophy as a theoretical foundation and methodological approach that focuses on the inherently interpretive processes in which a reader engages in an ever-expanding and deepening understanding of a relevant body of literature. Adopting a comprehensive literature review approach that addresses well-known issues resulting from applying structured literature review approaches (e.g., Webster & Watson, 2002), we strive toward the dual purpose of hermeneutic analysis – i.e., to synthesize and critically assess the body of knowledge (Boell & Cecez-Kecmanovic, 2014). We would like to emphasize that the hermeneutic approach to literature reviews is not in opposition to structured approaches. Rather, it addresses the weaknesses of structured approaches (i.e., that they view engagement with the literature as a routine task rather than as a process of intellectual development) and complements them with the hermeneutic perspective to create a holistic approach for conducting literature reviews.

### Theoretical underpinning and research method

**Fig. 1:**
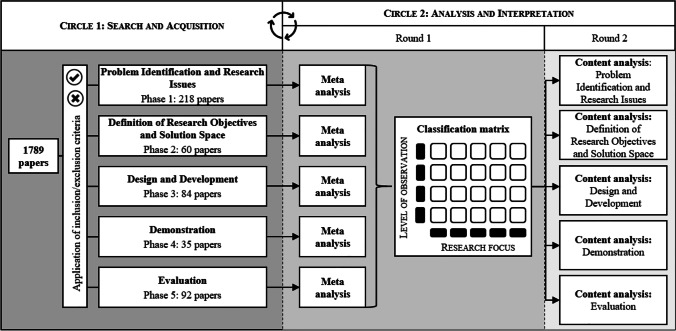
Hermeneutic procedure applied to the literature review

A methodological means for engaging in reciprocal interpretation of a whole and its constituent elements is the hermeneutic cycle (Bleicher, 2017), which consists of a mutually intertwined *search and acquisition circle* (Circle 1 in Fig. 1) and the wider *analysis and interpretation circle* (Circle 2 in Fig. 1) (Boell & Cecez-Kecmanovic, 2014). Figure 1 depicts the steps associated with the hermeneutic literature review. The search and acquisition circle is shown on the left of the figure, while the analysis and interpretation circle containing steps of meta and content analysis is depicted on the right. The two circles should be understood as an iterative procedure, the nature of which will be explained in the following.

### Circle 1: Search and acquisition

The hermeneutic literature review starts with the search and acquisition circle, which is aimed at finding, acquiring, and sorting relevant publications. In line with holistic thinking, we began with the identification of a rather small set of highly relevant literature (Boell & Cecez-Kecmanovic, 2014) and went on to identify further literature on the basis of progressively emerging keywords. This step is central to the hermeneutic approach and addresses a criticism on structured literature reviews, namely that they downplay the importance of reading and dialogical interaction between the literature and the reader in the literature search process, reducing it to a formalistic search, stifling academic curiosity, and threatening quality and critique in scholarship and research (Boell & Cecez-Kecmanovic, 2014; MacLure, 2005). Thus, while the search process remains formalized, as in pure structured approaches, the hermeneutic approach allows us to acquire more information about the problem at hand and to identify more relevant sources of information (Boell & Cecez-Kecmanovic, 2014).

Given our initial research question and the scope of the review, we began by searching for papers in the Association for Information System’s (AIS’s) eLibrary over a period of 30 years (1990 to 2019). We consider this database to be a source of the most significant publications in the field of HIS research with a focus on the IS research domain. Using the keywords ‘digital health’ and ‘digital healthcare service’, we identified an initial set of 54 papers based on the title, abstract, and keyword search. Engaging in a first round of the hermeneutic search and acquisition circle, we extended and refined these keywords by identifying emerging topics within the literature, as well as using backward and forward search (Webster & Watson, 2002). In particular, with each additional paper identified through backward and forward search, we compared keyword references in the papers to our list of keywords and added them if there was sufficient content delimitation. The decision to add a keyword was discussed with all authors until consensus was reached. This led us to a set of 12 keywords, including ‘electronic health’, ‘ehealth’, ‘mobile health’, ‘mhealth’, ‘health apps’, ‘tech health’, ‘healthcare services’, ‘healthcare informatics’, ‘medical informatics’, and ‘health data’.

The selection of publications being considered for our research comprised all journals belonging to the AIS eLibrary, the Senior Scholars’ Basket of Eight Journals (e.g., *European Journal of Information Systems, Information Systems Research*, and *MIS Quarterly*), well-regarded journals following the analyses of Chiasson and Davidson (2004) and Chen et al. (2019) (e.g., *Business & Information Systems Engineering*, *Communications of the ACM, and Decision Support Systems*), and the proceedings of the major AIS conferences (e.g., Americas Conference on Information Systems (AMCIS), International Conference on Information Systems (ICIS)). An overview of the selected journals and proceedings is provided in Appendix 1.

**Fig. 2:**
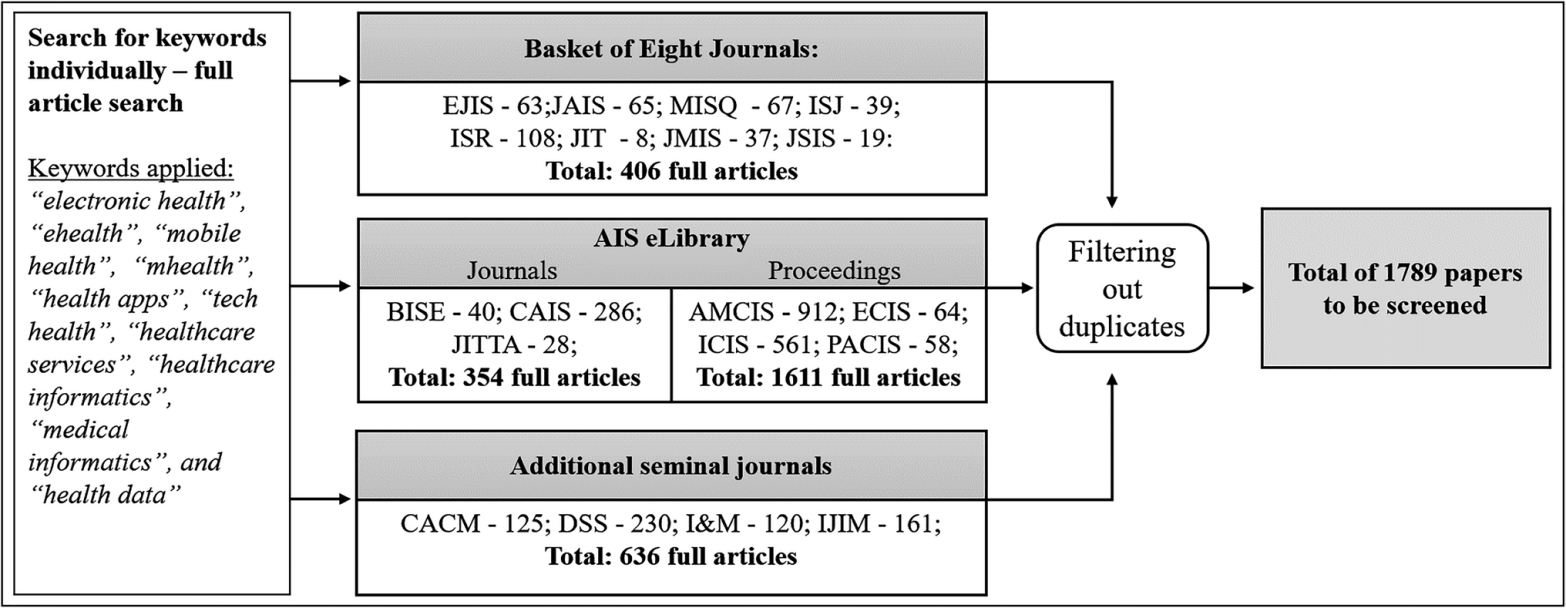
Steps of the search process to create the data set

Using our set of keywords, we searched for each keyword individually in the AIS eLibrary and the databases of the respective journals. Subsequently, we created a dataset and filtered out the duplicates, yielding a total number of 1,789 papers to be screened in the search and acquisition circle (Circle 1 in Fig. 1). Figure 2 provides an overview of this process by listing the total number of articles identified for each journal individually.

The resulting 1,789 papers progressively passed through the intertwined hermeneutic circles. Because of the large number, we divided the papers at random into four equally sized groups and assigned them to each of the authors. Each author then screened the paper in his or her group. In the course of several rounds of discussion, decisions on the inclusion of keywords and articles in the literature review were made by all authors, based on the original recommendations of the author responsible for the respective group. To ensure rigor and transparency of the analysis and results, we kept a logbook in which all decisions of the authors and steps of the literature review were recorded (Humphrey, 2011).

Given the abundance of topics that were already apparent from titles and abstracts, we began to sort the publications (Boell & Cecez-Kecmanovic, 2014). The process of sorting proved to be challenging, as HIS research is diverse and tends to be eclectic (Agarwal et al., 2010). This is why researchers have developed frameworks for clustering and analysing HIS research (LeRouge et al., 2007). So far, however, no consent on a unified framework has emerged, and sorting is often strongly influenced by the authors’ views on HIS research (Agarwal et al., 2010; Fichman et al., 2011). For instance, Agarwal et al. (2010) predetermined health IT adoption and health IT impact as major themes associated with health ITs, acknowledging that this pre-categorization of research topics made a systematic review of the growing and increasingly complex HIS literature unfeasible. Consequently, we decided to sort the articles we had identified into groups inspired by and loosely related to the phases of design science research (DSR) (Peffers et al., 2008), which is an essential step in hermeneutics – i.e., defining guidelines to facilitate interpretive explication (Cole & Avison, 2007). DSR can be understood as a cumulative endeavour and, therefore, we understood HIS research as accumulative knowledge that can be reconstructed and consolidated using DSR phases as guidance (vom Brocke et al., 2015; vom Brocke et al., 2009). In particular, this helped us to sort the articles without prejudice to expected HIS research topics and clusters (Grondin, 2016).

In the past, researchers have used the DSR process in the context of literature reviews to identify advances in design science-related research outcomes (Offermann et al., 2010). In this paper, we use the DSR phases – in the sense of a rough guideline – as a neutral lens to classify articles according to their research outcomes. We thereby assume that HIS literature can be seen as an overall process, where research results and progress are built upon each other and can be classified into phases of *problem identification and research issues*, *definition of research objectives and possible solution space*, *design and development of solutions*, *demonstration of research effectiveness, innovativeness and acceptance*, and *evaluation*. These phases served as a guide to achieve an outcome-oriented, first-hand sorting of articles, while this approach also gave us the opportunity to take a bird's-eye view on HIS research. Note that we intentionally omitted the last step of DSR – i.e., communication – as we regard communication as present in all published articles. Based on our initial reading, we assigned all 1,789 papers to the phases and discussed this sorting in multiple rounds until all authors agreed on the assignments.

Simultaneously, we applied criteria for the inclusion and exclusion of articles. We included full papers published in the journals and conference proceedings belonging to our selection. We excluded articles that were abstract-only papers, research-in-progress papers, panel formats, or workshop formats, as well as papers without direct thematic reference to our research objective. Additionally, during the acquisition stage we stored selected papers in a separate database whenever they fulfilled certain quality criteria (e.g., for separate studies using the same dataset, such as a conference publication and a subsequent journal publication, we only used the articles with the most comprehensive reporting of data to avoid over-representation).

The authors read the resulting 489 papers to identify new core terms and keywords that were used in subsequent searches, which not only provided the link to the analysis and interpretation circle but also informed the literature search. For this purpose, each author read the papers and kept notes in the logbook that supported us in systematically recording the review process and allowed us to shift from concentrating on particular papers to focusing on scientific concepts (Boell & Cecez-Kecmanovic, 2014; Webster & Watson, 2002).

### Circle 2: Analysis and interpretation

The search and acquisition circle formed part of the iterative procedure of analysis and interpretation, whereby the reading of individual papers was the key activity linking Circle 1 to the steps of Circle 2 (Boell & Cecez-Kecmanovic, 2014). Through orientational reading we gained a general understanding of the literature, thus laying the foundation for the subsequent steps of analysis and interpretation (Boell & Cecez-Kecmanovic, 2014).

Within the analysis and interpretation circle, two types of reviews were conducted for all identified and sorted articles: in a first round a meta-review, and in a second round a content analysis of the papers was performed. Meta-reviews are a useful tool for capturing and analysing massive quantities of knowledge using systematic measures and metrics. We followed Palvia et al. (2015), who proposed a structured method that is integrated into the hermeneutic approach. In particular, having identified and sorted the relevant research articles, we applied proposed review features, including methodological approach, level of observation, sample size, and research focus (Humphrey, 2011; Palvia et al., 2015) to map, classify, and analyse the publications (Boell & Cecez-Kecmanovic, 2014). In doing so, we slightly adapted the classic meta-analysis by focusing on meta-synthesis, which is similar to meta-analysis but follows an interpretive rather than a deductive approach. Whereas a classic meta-analysis tries to increase certainty in cause-and-effect conclusions, meta-synthesis seeks to understand and explain the phenomena of mainly qualitative work (Walsh & Downe, 2005). The results of the meta-synthesis provided the basis for our subsequent critical assessment of content. Furthermore, we created a classification matrix based on particularly salient features of the meta-review (i.e., levels of observation and research foci), which facilitated and standardized the content analysis.

Within the matrix, the *levels of observation* comprised infrastructure (e.g., information exchange systems, electronic health records), individuals (patients and users of digital health services), professionals (e.g., nurses and general practitioners), organizations (hospitals and other medical institutions), and an ecosystem level. The latter is defined as individuals, professionals, organizations, and other stakeholders integrated via a digital infrastructure and aiming to create a digital environment for networked services and organizations with common resources and expectations (Leon et al., 2016). To identify the most important concepts used by researchers, we discussed a variety of approaches to the derivation of *research foci* – i.e., areas containing related or similar concepts that are frequently used in research on HIS. Finally, six research focus areas emerged, covering all relevant research areas. To describe the core HIS research issues addressed by these foci, we used the following questions:

- HIS strategy: *What are the prerequisites for configuring, implementing, using, maintaining, and finding value in HISs?*
- HIS creation: *How are HISs composed or developed?*
- HIS implementation: *How are HISs implemented and integrated?*
- HIS use and maintenance: *How can HISs be used and maintained once in place?*
- Consequences and value of HIS: *What are the consequences and the added value of HISs?*
- HIS theorization: *What is the intellectual contribution of HIS research?*

We used the classification matrix as a tool for assigning publications and finding patterns across research articles and phases. In particular, we used open, axial, and selective coding (Corbin & Strauss, 1990) to analyse the content of articles in a second round of the analysis and interpretation circle. Each author individually assigned open codes to text passages while reading the identified research articles, noting their thoughts in the shared digital logbook that was used for constant comparative analysis. Once all authors had agreed on the open codes, axial coding – which is the process of relating the categories and subcategories (including their properties) to each other (Wolfswinkel et al., 2013) – was conducted by each author and then discussed until consent on codes was reached. Next, we conducted selective coding and discussed the codes until theoretical saturation was achieved (Corbin & Strauss, 1990; Matavire & Brown, 2008). For the sake of consistent terminology, we borrowed terms from Chen et al. (2019), who used multimethod data analysis to investigate the intellectual structure of HIS research. In particular, they proposed 22 major research themes, which we assigned to the initial codes whenever possible. In two rounds of discussion in which we compared the assignment of codes, two additional codes emerged, which left us with a total of 24 theme labels (Appendix 2). By discussing the codes at all stages of coding, theoretical saturation emerged, which is the stage at which no additional data are being found or properties of selective codes can be developed (Glaser & Straus, 1968; Saunders et al., 2018). In fact, independent from each other, all authors saw similar instances occurring over and over again, resulting in the same codes, making us confident that we had reached theoretical saturation (Saunders et al., 2018).

Finally, we entered the codes into the classification matrix, which allowed us to identify patterns based on the meta and content analysis. This enabled us to provide insights into the strengths and weaknesses of current HIS research; these are presented in the following section.

## Zooming-in: key findings of the phase-based literature analysis

In the following, we ‘zoom in’ (Gaskin et al., 2014) by presenting key findings of the literature review for each phase, illustrated by means of the classification matrices. We assigned selective codes that emerged from the content analysis to the fields of the matrices, with the numbers in brackets indicating the frequency with which codes emerged. Note that, for the sake of clarity, we displayed only the most relevant research themes in the matrices and indicated the number of further papers using the reference ‘other themes.’ A complete list of research themes for each phase can be found in the appendix (Appendix 2). In the following, each table shows the classification matrix and selective codes that resulted from the meta and content analysis of papers in the respective phase. The shaded areas in the matrix show focused research themes (i.e., selective codes) and characteristics of research articles that gave way to clusters (i.e., collections of themes that appear frequently and/or characteristically for the respective focus).

### Phase 1: Problem identification and research issues

Within the first phase, a large body of literature was found (218 articles). This phase encompasses articles that identify problems and novel research issues as a main outcome, with the aim of pointing out shortcomings and provoking further research. For instance, besides behavioural issues such as missing user acceptances or trust in certain HISs, the design and effectiveness of national health programs and/or HIS is a frequently mentioned topic. It should be noted, however, that literature assigned to this phase is extremely diverse in terms of research foci, levels of observation, and research themes, and hardly any gaps can be identified (Table 1).

**Table 1:**
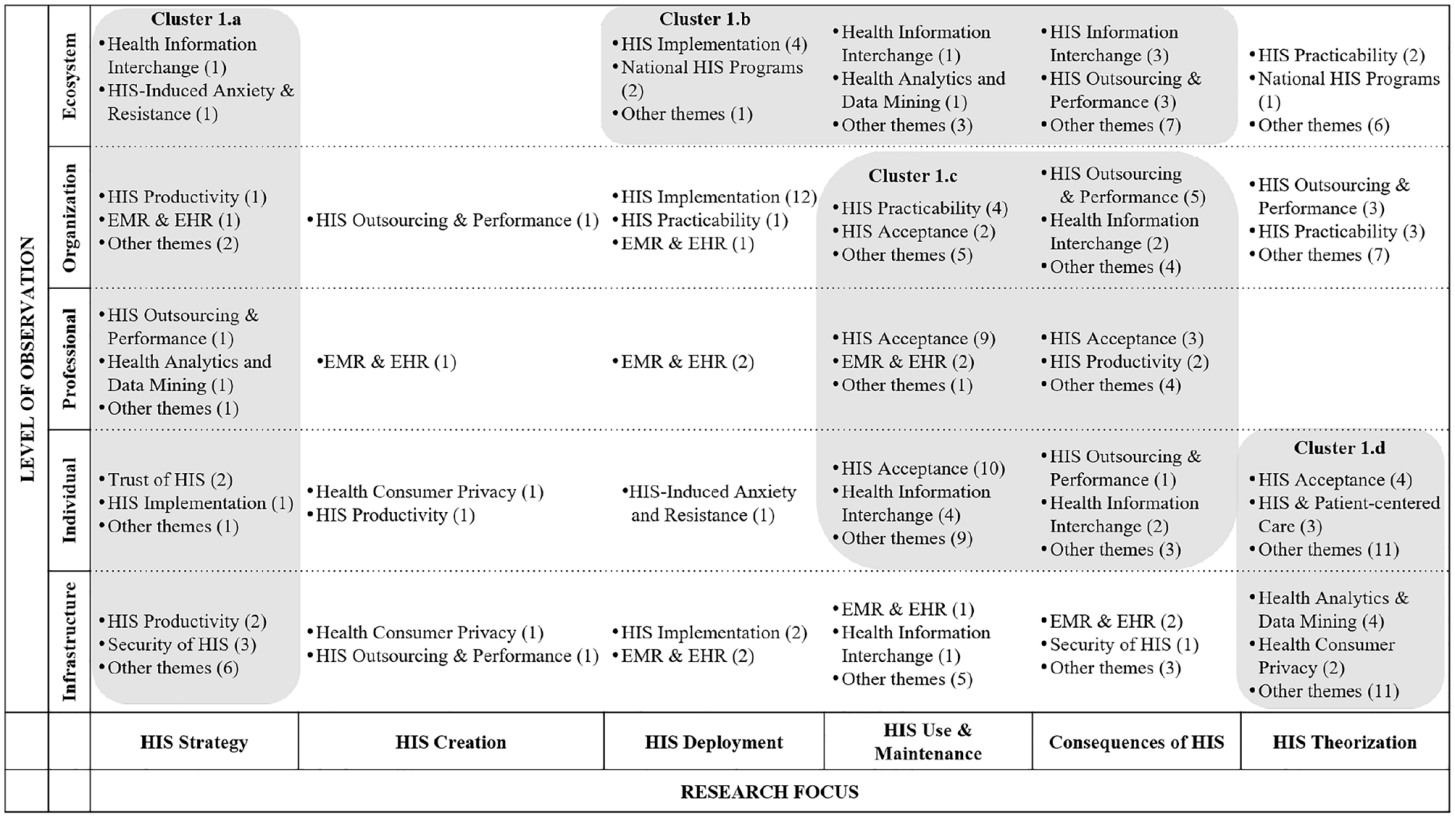
HIS publications assigned to the phase of problem identification and research issues

The first cluster (1a) encompasses the research focus of HIS strategy, spanning all levels of observation and totalling 24 publications. HIS strategy appears to be of particular relevance to the levels of organization and infrastructure. Content-wise, the theme of health information interchange is of particular interest, referring, for example, to the development of a common data infrastructure (Ure et al., 2009), consumer-oriented health websites (Fisher et al., 2007), and security risks of inter-organizational data sharing (Zhang & Pang, 2019). HIS productivity and HIS security are the second most salient themes, focusing, for example, on measuring the effectiveness of fitness apps (Babar et al., 2018) and presenting challenges with regard to the interoperability of medical devices (Sametinger et al., 2015).

The second cluster (1b), comprising 25 publications, represents the ecosystem level and focuses mainly on national and cross-national HIS-related issues such as the relation between ICT penetration and access to ehealth technologies across the European Union (Currie & Seddon, 2014), as well as on the collaboration and involvement of different stakeholders (Chang et al., 2009; King, 2009). Most important here is health information interchange – e.g., the provision, sharing, and transfer of information (Bhandari & Maheshwari, 2009; Blinn & Kühne, 2013).

Cluster 1c covers the research focus of HIS use and maintenance, as well as the consequences of HIS. Whereas most papers addressing the HIS acceptance theme focus on professionals’ or patients’ acceptance of specific technological solutions, such as telemedicine (Djamsbi et al., 2009) or electronic health records (Gabel et al., 2019), papers assigned to health information interchange focus on topics related to information disclosure, such as self-tracking applications (Gimpel et al., 2013). Finally, the HIS outsourcing and performance theme concentrates on financial aspects in organizations, including potential for quality improvements and cost reductions (Setia et al., 2011; Singh et al., 2011).

Finally, the fourth cluster (1d) focuses on HIS theorizing with respect to the individual and infrastructure levels of observation. Although this cluster represents a range of theme labels (15), those addressing HIS acceptance, HIS patient-centred care, as well as health analytics and data mining predominate. Papers within the theme label HIS acceptance cover a wide range of topics, such as the acceptance of telehealth (Tsai et al., 2019) up to the usage intentions of gamified systems (Hamari & Koivisto, 2015). The same applies to the papers assigned to the theme labels of health analytics and data mining. Focusing on the infrastructure level of observation, the identified papers mostly review academic research on data mining in healthcare in general (Werts & Adya, 2000), through to the review of articles on the usage of data mining with regard to diabetes self-management (Idrissi et al., 2019). Papers on HIS patient-centred care mostly address the challenges and opportunities of patient-centred ehealth applications (Sherer, 2014).

Apart from these clusters, quite a few research articles refer to the infrastructure level of observation, addressing information sharing in general (Li et al., 2008), electronic medical records (George & Kohnke, 2018; Wessel et al., 2017), and security and privacy issues (Zafar & Sneha, 2012).

Most common in terms of research methods within this phase are case studies (57), followed by quantitative data analyses (50), theoretical discussions (29), and literature studies (14). In particular, case studies dominate when referring to the ecosystem or infrastructure level of observation, whereas quantitative analyses are conducted when individuals or professionals are at the centre of the discussion. However, and unsurprisingly given the considerable diversity of research themes within this phase, the variety of research methods is also quite large, ranging from field studies (Paul & McDaniel, 2004), to interviews (Knight et al., 2008), to multimethod research designs (Motamarri et al., 2014).

### Phase 2: Definition of research objectives and solution space

The second phase of HIS research yielded a lower number of articles (45) compared to the phase of problem identification and research issues. The second phase comprises articles that focus on proposing possible solutions to existing problems – i.e., introducing theory-driven, conceptual designs of health ecosystems including health information interchange, as well as scenario analyses anticipating the consequences of HIS implementation on an organizational level. Based on the research foci and levels of observation, we identified three specific thematic clusters, as shown in Table 2.

**Table 2:**
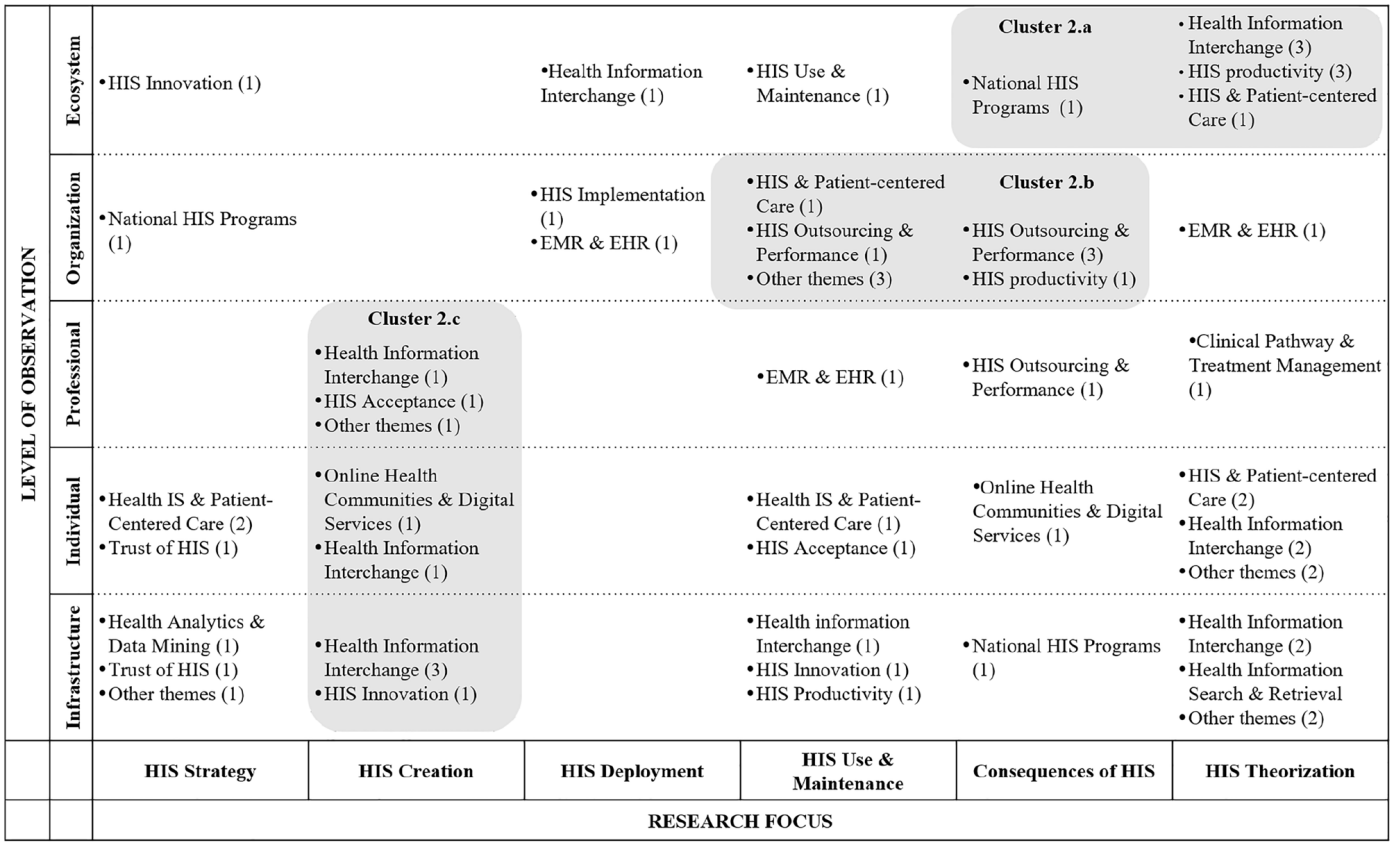
HIS publications assigned to the definition of research objectives and solution space

The first cluster (2a) comprises the ecosystem level of observation and encompasses eight publications. Besides a strong tendency toward theory-driven research, health information interchange is the most common theme. We found that the need to enable cooperation within networks and to ensure accurate data input was addressed in most of the literature. While a majority of studies focus on the application of HIS in networks within specific boundaries, such as medical emergency coordination (Sujanto et al., 2008) or Singapore’s crisis management in the fight against the SARS outbreak in 2003 (Devadoss & Pan, 2004), other studies, such as that by Aanestad et al. (2019), take an overarching perspective, addressing the need to break down silo thinking and to start working in networks. Following the question of why action research fails to persist over time, Braa et al. (2004) highlighted the role of network alignment, criticizing action research projects for failing to move beyond the prototyping phase and, therefore, failing to have any real impact.

Cluster 2b, encompassing nine publications, was derived from the observation that studies within the organizational level concentrated strongly on HIS use and maintenance and the consequences of HIS research. Herein, a vast array of topics was observed, such as the potential for cost reduction through HIS (Byrd & Byrd, 2009), the impact of HIS on product and process innovation in European hospitals (Arvanitis & Loukis, 2014), and the perceived effectiveness of security risk management in healthcare (Zafar et al., 2012). Moreover, we found that practice-oriented methods, such as mixed-method approaches, surveys, data analyses, and case studies, are used predominantly within this cluster. Focusing on the latter, most studies analyse particular scenarios by using a rather small sample of cases, for instance, Al-Qirim (2003) analysed factors influencing telemedicine success in psychiatry and dermatology in Norway.

The third cluster (2c) was derived from analysis of the HIS creation research focus (nine publications). Although health information interchange is the most represented in this cluster, a large number of further themes can be observed. Studies within this cluster predominantly address design aspects of system interoperability, focusing on data processing and data interchange between the actors. HISs mostly serve as a tool for the development or enhancement of decision support systems, such as for real-time diagnostics combining knowledge management with specific patient information (Mitsa et al., 2007) or clinical learning models incorporating decision support systems in the dosing process of initial drug selection (Akcura & Ozdemir, 2008).

### Phase 3: Design and development

The design and development phase comprises 84 research articles concerned with the creation of novel IS artefacts (e.g., theories, models, instantiations). We thereby refer to Lee et al.’s (2015) definition of the IS artefact – i.e., the information, technology, and social artefact that forms an IS artefact by interacting. We assigned to this phase papers that are explicitly concerned with developing solutions for information exchange (e.g., design of messaging systems or knowledge systems in hospitals), technological artefacts (e.g., hardware or software used for generating electronic health records), and social artefacts that relate to social objects (e.g., design of national or international institutions and policies to control specific health settings and patient-centred solutions). Within the design and development phase, the analysis revealed two clusters (Table 3).

**Table 3:**
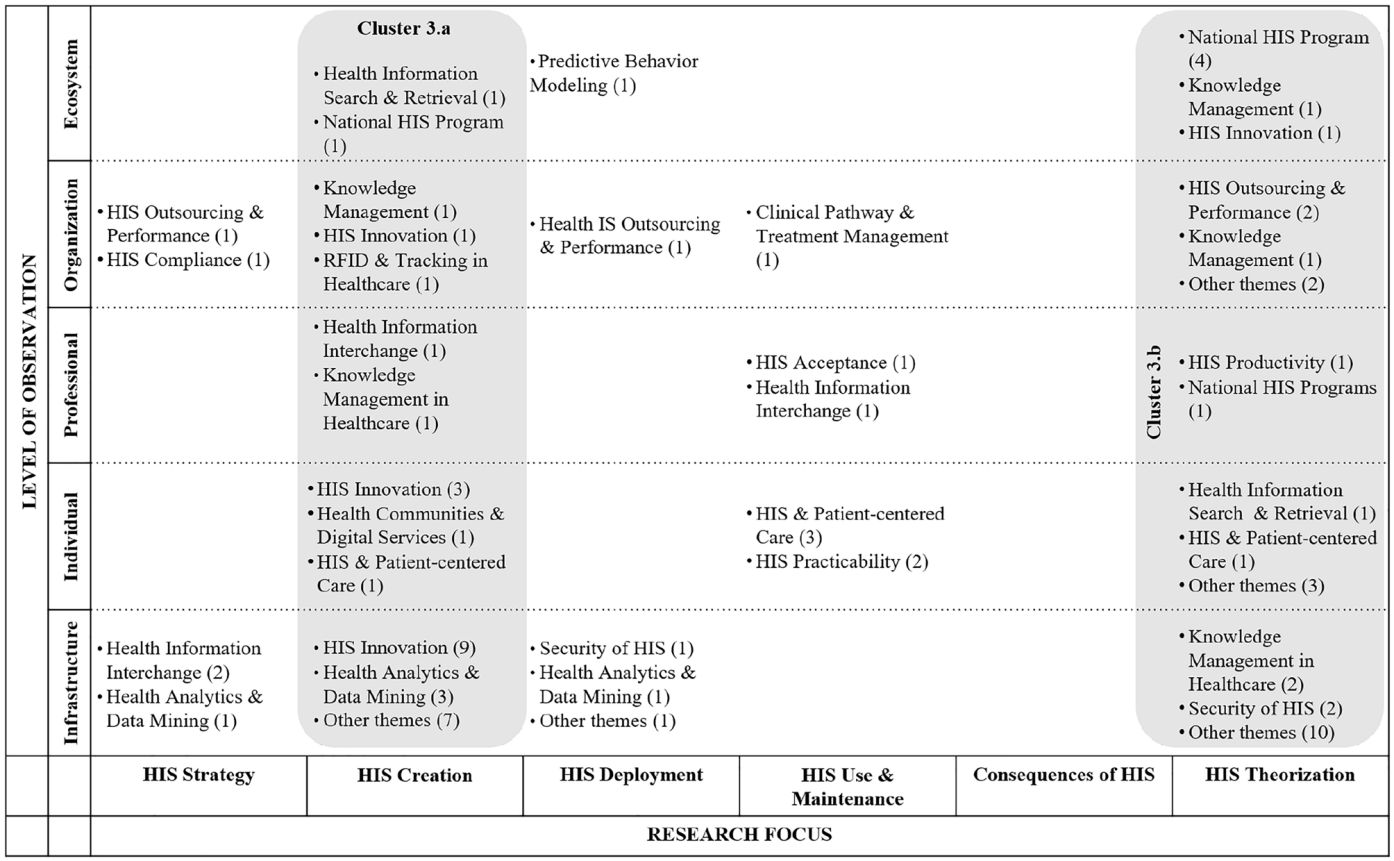
HIS publications assigned to the design and development phase

The first cluster (3a) was identified in the research focus of HIS creation (31 articles). Here, the most frequent research theme is HIS innovation followed by HIS and patient-centred care, HIS productivity, and health analytics and data mining. The focus is on specific contexts, mostly medical conditions and artefacts developed for their treatment, such as in the context of mental health/psychotherapy (Neben et al., 2016; Patel et al., 2018), diabetes (Lichtenberg et al., 2019), or obesity (Pletikosa et al., 2014). Furthermore, information infrastructures or architectures – for instance, for the process of drug prescription (Rodon & Silva, 2015), or for communication between healthcare providers and patients (Volland et al., 2014) – are represented.

The second aggregation of research articles is found in cluster 3b, focusing on theoretical aspects of HIS (32 articles). Again, these studies span all levels of observation (including infrastructure, individual, professional, organization, and ecosystem). Topics in this theme are diverse, ranging from HIS on a national level (Preko et al., 2019), to knowledge management in healthcare (Wu & Hu, 2012) to security of HIS (Kenny & Connolly, 2016).

Beyond both clusters, it is evident that during design and development, researchers do not deal with the consequences of HIS, nor does HIS strategy play an important role. Furthermore, only in the research focus of theorization is the ecosystem level of some relevance to other levels (e.g., the individual level). It should be noted that ecosystems are mostly referred to in terms of nations or communities, without any transnational or global perspective. Furthermore, the term ‘ecosystem’ has not been used in research, and within the other research focus areas, the ecosystem level is barely represented. Moreover, articles combining different perspectives of the single levels of observation on HIS – namely individuals (i.e., patients), professionals (i.e., medical staff), and organizations (e.g., hospitals) – are rare. During design and development, potential users are not typically integrated, whereas it is quite common to derive requirements and an application design from theory, only involving users afterwards – e.g., in the form of a field experiment (e.g., Neben et al., 2016).

Surprisingly, theoretical papers outweigh papers on practical project work, whereby the latter mostly focus on a description of the infrastructure or artefact (e.g., Dehling & Sunyaev, 2012; Theobalt et al., 2013; Varshney, 2004) or are based on (mostly single) case studies (e.g., Hafermalz & Riemer, 2016; Klecun et al., 2019; Ryan et al., 2019). Within the design and development phase, the generation of frameworks, research models, or taxonomies is prevalent (e.g., Preko et al., 2019; Tokar et al., 2015; Yang & Varshney, 2016).

### Phase 4: Demonstration

This phase includes 35 articles related to presenting and elaborating on proposed solutions – e.g., how HIS can be implemented organization-wide (e.g., via integration into existing hospital-wide information systems), proposed strategies and health policies, as well as novel solutions that focus on health treatment improvements. Within the demonstration phase, we identified two clusters that emerged from the meta and content analyses (Table 4).

**Table 4:**
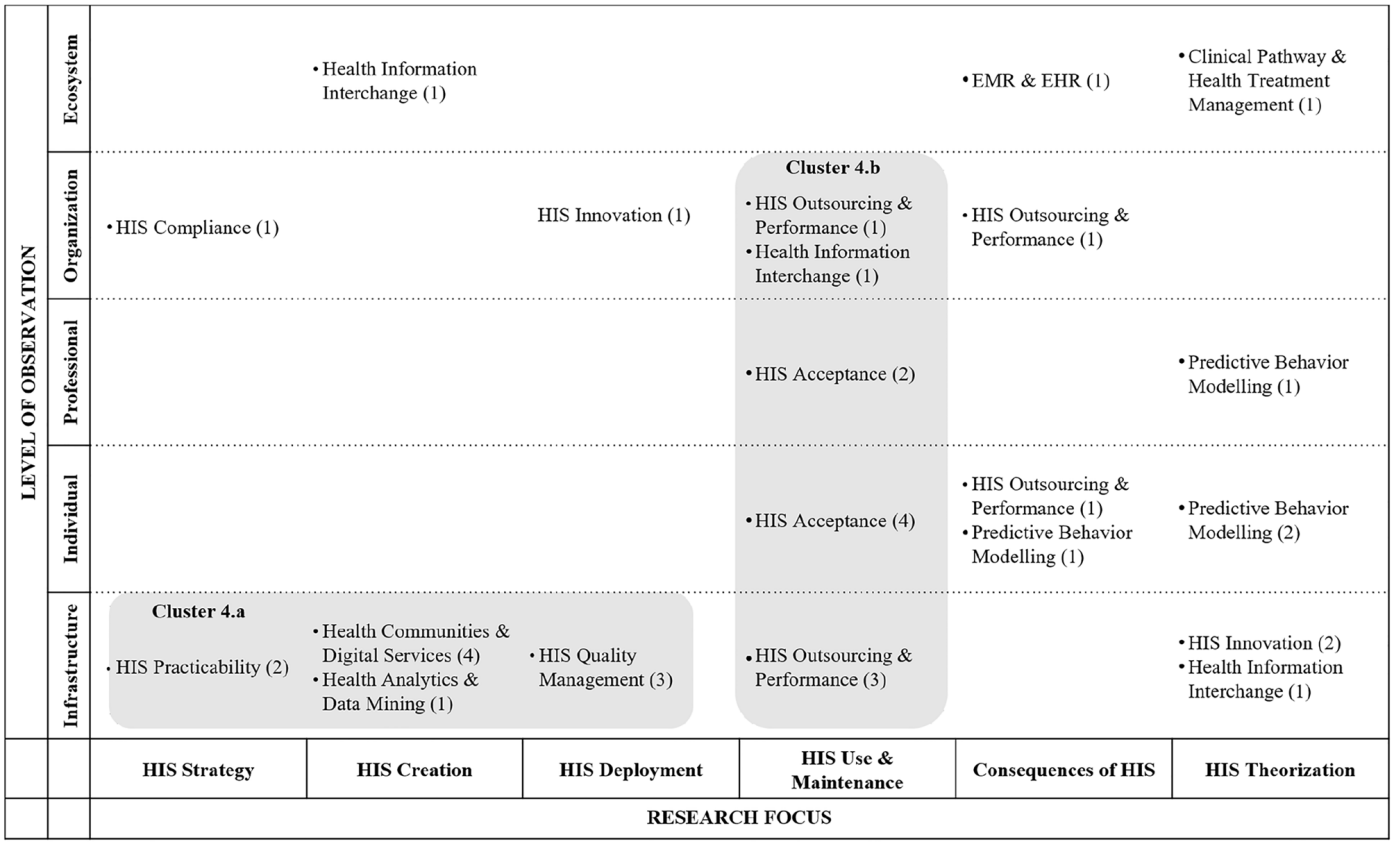
HIS publications assigned to the demonstration phase

Cluster 4a (10 articles) is characterized by articles that focus on HIS issues related to the infrastructure level, spanning the research foci of HIS strategy, creation, and deployment. Content-wise, the cluster deals mainly with technical feasibility and desirability of HISs, including topics such as the configuration of modular infrastructures that support a seamless exchange of HISs within and between hospitals (Dünnebeil et al., 2013). Moreover, papers in this cluster address HIS practicability by determining general criteria that are important for the design of health information systems (Maheshwari et al., 2006) or conduct HIS application tests by carrying out prototypical implementations of communication infrastructures. In particular, the latter are tested and proven to meet specific technical standards to guarantee the frictionless transmission of health information data (Schweiger et al., 2007). In contrast, Heine et al. (2003) upscaled existing HIS solutions and tested the infrastructure in large, realistic scenarios.

Conversely, cluster 4b (11 articles) is mainly concerned with HIS use and maintenance, spanning several levels of observation – i.e., infrastructure, individuals, professionals, and organizations. Interestingly, papers in this cluster aim at efficiency and added value when looking at the infrastructure and organizational levels, whereas researchers are more interested in acceptance when focusing on the individual and professional use of HISs. Overall, cluster 4b is primarily concerned with organizational performance (e.g., increases in efficiency due to better communication and seamless transfer of patient health information) as well as user acceptance of new HISs.

Although the two clusters constitute a diverse set of literature and themes, it is apparent that research taking an ecosystem perspective is very rarely represented. Across the papers, only three are concerned with issues related to the ecosystem level. In particular, Lebcir et al. (2008) applied computer simulations in a theoretical demonstration as a decision support system for policy and decision-makers in the healthcare ecosystem. Abouzahra and Tan (2014) used a mixed-methods approach to demonstrate a model that supports clinical health management. Findikoglu and Watson-Manheim (2016) addressed the consequences of the implementation of electronic health records (EHR) systems in developing countries.

### Phase 5: Evaluation

The fifth phase includes 92 publications with a focus on assessing existing or newly introduced HIS artefacts – i.e., concepts, policies, applications, and programs – thereby proving their innovativeness, effectiveness, or user acceptance. As Table 5 shows, three clusters were identified.

**Table 5:**
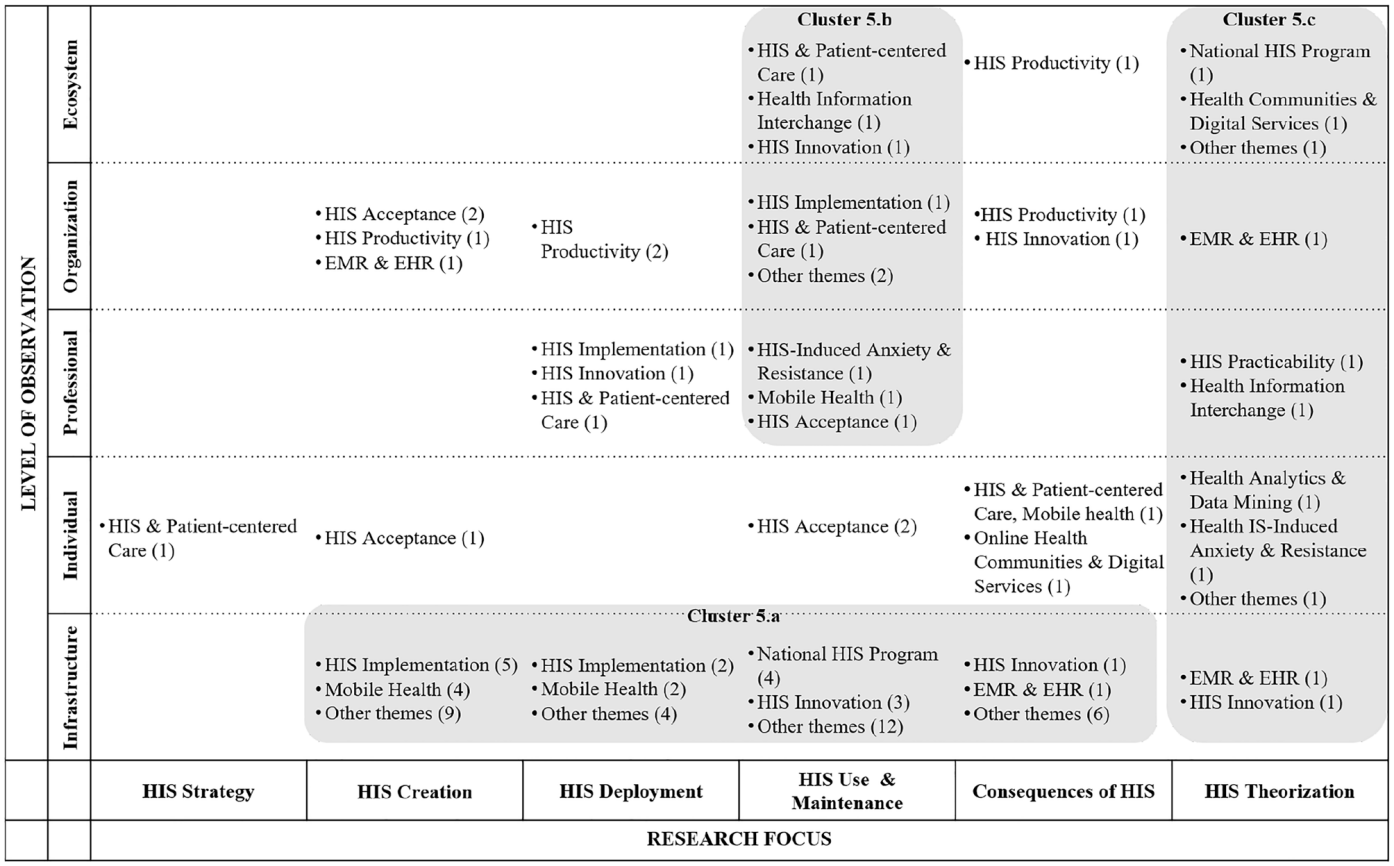
HIS publications assigned to the evaluation phase

The main focus of publications in the evaluation phase is on the infrastructure level, where most papers are related to HIS creation and HIS use and maintenance. Therefore, together with the publications pigeonholed to HIS deployment and consequences of HIS, these articles were summarized as the first cluster (5a, comprising 53 articles). The assessment of national HIS programs, as well as mobile health solutions, are a frequent focus (10 papers). Articles on HIS use and maintenance are largely related to the professional, organizational, and ecosystem levels and were thus grouped as cluster 5b (10 articles). A third cluster (5c – 11 articles) emerged from research articles in HIS theorization. Here, papers at all levels of observation were found. Research focusing on areas such as HIS strategy and consequences of HIS are, with a few exceptions, not covered in the evaluation phase. Methods used include interviews, focus groups, and observations (e.g., Romanow et al., 2018). Experiments and simulation are rarely applied (e.g., Mun & Lee, 2017). The number of interviews shows a huge spread, starting with 12 and reaching a maximum of 150 persons interviewed.

Under the evaluation lens, the ecosystem perspective is covered by seven articles, but only three papers look at cases, while the others focus on theorization or consequences in terms of costs. Overall, popular topics in the evaluation phase include mobile health and the fields of electronic medical records (EMR) and EHR, e.g., Huerta et al. (2013); Kim and Kwon (2019). The authors cover these themes mostly from an HIS creation perspective; thus, they deal with concrete concepts, prototypes, or even implemented systems. In the evaluation phase, just nine papers deal with HIS innovation – a good example being Bullinger et al. (2012), who investigated the adoption of open health platforms. We may conclude that, in most cases, evaluation is related to more established technologies of HIS. As expected, most articles in this phase rely on practice-oriented/empirical work (as opposed to theory-driven/conceptual work). Just two papers (Ghanvatkar & Rajan, 2019; Lin et al., 2017) deal with health analytics and data mining, one of the emerging topics of HIS.

## Zooming out: key findings of the literature analysis across phases

Having elaborated on the key findings within each phase of HIS research, we now ‘zoom out’ (Benbya et al., 2020; Gaskin et al., 2014) to recognize the bigger picture. Thereby, we ‘black-box’ the concrete research themes (e.g., HIS implementation, health analytics, HIS innovation) to focus on clusters across phases, highlighting the breadth that HIS research encompasses (Leroy et al., 2013). In particular, while we focused on analysing the main topics within the different phases of HIS research in the zoom-in section, we now abstract from those to perform a comparative analysis of emerging clusters across those phases by zooming out. We do so by comparing the different clusters, taking into account the aspects of the level of observation and the research foci, which gave us the opportunity to identify areas of strong emphasis and potential gaps.

In particular, each author first conducted this comparative analysis on their own and then discussed and identified the potential weaknesses together. This was done in two rounds of discussion. In particular, it became obvious which areas hold immense potential for further research in healthcare (especially the penetration of new, initially non-healthcare actors, such as tech companies or other providers pushing into the industry). We summarize these potentials for research by proposing four pathways that can help HIS research to broaden its focus so that we can better understand and contribute to current developments. Notably, we expect that these insights will help to assess the state-of-the-art of HIS research and its preparedness for dealing with the consequences of Covid-19 and further pandemics, as well as for coping with associated exogenous shocks.

In zooming out, we identified discrepancies between phase 1 (problem identification and research issues) and the subsequent phases. In particular, the diversity of topics was considerably lower when it came to how researchers determined strategies; created, demonstrated, used, and maintained HISs; and coped with the consequences thereof. We observed that researchers pointed to a diverse set of issues that span all levels of observation, especially in HIS theorization, focusing on topics such as trust in HIS, data analytics, and problems associated with the carrying out of national health programs. Surprisingly, although we can assume that researchers recognized the multidimensionality of issues as a motivation to conduct HIS research, they did not seem to approach HIS research issues in a comprehensive and consistent way.

To illustrate this assertion, we point to the ‘shift of clusters’ that can be observed when comparing the single phases, from problem identification to the evaluation of HIS. We note that clusters increasingly migrate ‘downwards’ (i.e., from the ecosystem level down to the infrastructure level) and become even fewer. In line with Braa et al. (2004), we suggest that extant HIS research has identified a multitude of interrelated issues but has faced problems in translating these approaches into concrete and holistic solutions. This is reflected in the lower number of, and reduced diversity in, clusters across research themes when we move through the HIS research phases. Thus, we conclude that future HIS research can be broadened by taking into account the following pathway:

**HIS research is well-prepared and able to identify and theorize on systemic problems related to the healthcare industry. Nonetheless, it has the potential to address these problems more thoroughly – i.e., to find solutions that are as diverse as the problems and, thus, suitable for coping with issues in the healthcare industry characterized by the involvement of multiple actors, such as governments, healthcare providers, tech companies, and their interactions in diverse ecosystems (pathway 1).**

As we have seen, HIS research has tended to focus on important but incremental improvements to existing infrastructures, particularly in the phases of demonstration and evaluation, with the aim of presenting new IS artefacts and conceptual or practical solutions. For instance, Choi and Tulu (2017) considered improvements in user interfaces to decrease the complexity of mobile health applications using incremental interface design changes and altering touch techniques. Similarly, Roehrig and Knorr (2000) designed patient-centred access controls that can be implemented in existing infrastructures to increase the privacy and security of EHRs and avoid malicious access and misuse of patient health information by third parties.

While we sincerely acknowledge these contributions and wish to emphasize the multitude of papers that are concerned with enhancements to existing infrastructures, we would like to shift the view to the major challenges in HIS research. These challenges include combating global and fast-spreading diseases (e.g., malaria, tuberculosis, Covid-19) and tracking health statuses accurately and efficiently, especially in developing countries. All of these challenges necessitate global and comprehensive solutions, spanning individuals, organizations, and nations, and have to be embedded in a global ecosystem (Winter & Butler, 2011). Such grand challenges are, by nature, not easy to cope with, and the intention to develop a comprehensive solution from the perspective of IS researchers seems almost misguided. However, HIS research is currently missing the opportunity to make an impact, despite the discipline’s natural intersection with essential aspects of the healthcare industry (i.e., its infrastructures, technologies, and stakeholders, and the interdependencies between these components). Thus, we assert that:

**HIS research has often focused on necessary and incremental improvements to existing IS artefacts and infrastructures. We see potential in shifting this focus to developing solutions that combine existing IS artefacts to allow for exchange of information and the creation of open systems, which will enhance support for and understanding of the emergence of ecosystems (pathway 2).**

By focusing on incremental improvements, HIS research has become extraordinarily successful in solving isolated issues, especially in relation to the problems of patients and health service providers (e.g., hospitals and general practitioners). However, we observed during our analysis that spillover effects were seldom investigated. When, for example, a new decision support system in a hospital was introduced, positive consequences for patients, such as more accurate diagnoses, were rarely of interest to the research. In fact, our meta-analysis revealed that the level of observation for the majority of papers matched the level of analysed effects. While it is valid to investigate productivity and efficiency gains by introducing a hospital-wide decision support system, we are convinced that spillover effects (for instance, on patients) should also be within the focus of HIS research. Therein, we suggest that HIS research has not focused primarily on patients and their well-being but on IS infrastructures and artefacts. However, patient well-being is the ultimate direct (or indirect) goal of any HIS research (by increasing the accuracy and shortening the time of diagnosis, improving treatment success rates, etc.). Thus, we propose that:

**HIS research is experienced in solving isolated issues related to the daily processes of healthcare providers; however, we see much potential in considering the value that is delivered by focusing on patient-centricity (pathway 3).**

Putting the patient at the centre of HIS research implies shifting the focus of researchers to the patient’s own processes. The question remains as to how HIS researchers can support patient-centricity. While this is only possible by understanding patients’ processes, we also see the need to understand the whole system – i.e., the ecosystem in which patients’ processes are embedded. The ecosystem perspective needs to consider networked services and organizations, including resources and how they interact with stakeholders of the healthcare industry (including patients). To date, we observe, across phases the ecosystem perspective has largely been neglected. To be precise, although HIS research seems to be aware of the multilevel aspects of healthcare issues in the problem identification phase, researchers appear to stop or are hindered from developing solutions that go beyond the development of prototypes (Braa et al., 2004). Thus, we find that:

**HIS research is capable of theorizing on an ecosystem level (i.e., capturing the complexity of the socio-technical health system), but would benefit from increasing the transfer of these insights into research so as to develop holistic solutions (pathway 4).**

Looking at the strengths of HIS research, the reviewed papers accentuate the unique contribution that IS researchers can make to better understand and design IS artefacts for the healthcare context. This has been achieved by analysing empirical data and exploring contextual influences through the application and elaboration of IS theories (LeRouge et al., 2007). At the same time, our literature review shows the incredible diversity and high level of complexity of issues related to HISs, indicating that we need solutions characterized by holism and the inclusion of multiple actors (i.e., an integrative ecosystem perspective). So far, by concentrating on incremental improvements to existing infrastructures HIS research has widely failed to reach the necessary holistic level.

We would like to emphasize that we recognize the value of all previous approaches. Yet, it is necessary to ask whether we as IS researchers are in a position to identify current developments in the healthcare industry and to anticipate the consequences triggered by pandemics or other waves of disease. We acknowledge that this will be difficult unless we take a more holistic view and try to understand connections in the health ecosystems. Regarding whether HIS research is in a position to capture and anticipate consequences of the current push of tech companies in the healthcare industry catalysed, for example, by Covid-19, we assert that this is hardly the case, even if IS research is well-placed to interpret the expected socio-technical changes and adaptations within healthcare. Given the enormous potential for disruption caused by, for instance, pandemics and its consequences, such as the intrusion of technology companies into the market, it is now time to question and redefine the role of HIS research so that it can generate decisive impacts on the developments in this industry.

## Research agenda

To support HIS research for the transformation of the healthcare industry, we develop a research agenda that is informed by complexity theory. This theory implies that complex, socio-technical systems such as the healthcare industry can fluctuate between different states, ranging from homogenous forms of coevolution (i.e., a state where emergent structures and processes become similar to each other) to chaotic systems that are characterized by increasing levels of tension, which might result in extreme outcomes such as catastrophes or crises (Benbya et al., 2020).

While coevolution and chaos represent possible extreme states, the current situation – i.e., the penetration of tech companies into the healthcare industry – is best described by the dynamic process of emergence. Emergence is characterized by a disequilibrium, which implies unpredictability of outcomes that may lead to new structures, patterns, and properties within a system characterized by self-organization and bursts of amplification (Benbya et al., 2020; Kozlowski et al., 2013). Given the dynamics resulting from this, it seems impossible to predict the future; however, it is not impossible to prepare for it.

In particular, the current dynamics within the healthcare industry necessitate an understanding of exponential progress, not as the ability to foresee well-defined events in space and time, but as an anticipation of the consequences of emerging states and dynamic adaptive behaviours within the industry (Benbya et al., 2020). The following research agenda for HIS research is thus structured along three key issues: anticipating the range of actors’ behaviours, determining boundaries and fostering collaboration in the healthcare industry, and creating sustainable knowledge ecosystems.

According to these key issues, Table 6 offers guiding questions for HIS researchers. Addressing all issues will contribute to an understanding of the entire healthcare industry and the development of holistic solutions for a multitude of health issues by involving different actors (e.g., patients, hospitals, professionals, governments, NGOs). However, we propose approaching the agenda stepwise, in the order of the key issues, first looking at the range of behaviours and consequences of current developments for actors, then focusing on the blurring lines of the healthcare industry, and finally investigating the dissemination and sharing of knowledge, which we see as the ultimate means to connect actors and infrastructures to create a joint ecosystem. Table 6 thereby provides key guiding statements and exemplary research questions for future HIS research that support researchers in taking one of the aforementioned pathways. We structured guiding statements along three major areas of improvement. In addition, we offer exemplary research questions to these statements, as well as inspiring studies from other industries that have faced similar challenges and have been studied and supported by researchers.

**Table 6:** Agenda for a comprehensive research approach for future HIS-research

Areas of improvement	Guiding statements for future HIS research	Exemplary research questions and exemplary studies	Pathway
Anticipate the range of actor behaviours	• Determine existing and emergent actors in the healthcare industry • Identify actors’ interests and strategies in joining or staying in the healthcare industry • Align interests of established and new actors to achieve patient-centricity	• Why do companies engage in activities that lead to the blurring of industry boundaries (Nicholls-Nixon & Jasinski, 1995)? • Does the nature of boundary-crossing actions differ between firms (i.e., what is the value driver that leads companies into the healthcare industry) (Akkerman & Bakker, 2011)? • Does the infiltration of new actors into the healthcare system promote organizational learning for the benefit of the patient (Rivard et al., 2006)?	Pathway 1, Pathway 3
Determine boundaries and foster collaboration	• Capture and describe the blurring boundaries of the healthcare industry • Harness the advantages of blurring industry boundaries by proposing and developing appropriate ecosystem infrastructures • Create open systems that enable radical digital innovation	• How, and to what extent, do deregulation, globalization, and breakthroughs in science and information technology contribute to the blurring of boundaries (Rycroft & Kash, 2004)? • How can we leverage multi-company ecosystems with varying value propositions (Schwetschke & Durugbo, 2018)? • How is cross- and inter-industry innovation fostered, such as through absorptive potential or creative imitation (Enkel & Gassmann, 2010)?	Pathway 2
Create sustainable knowledge ecosystems	• Design and maintain permeable knowledge management systems for information interchange between actors in the healthcare industry • Implement structures of mutual benefit while protecting intellectual property rights • Ensure patient privacy while creating systems that allow for trustworthy predictions	• How should (knowledge and data) management systems be designed in order to balance information provision and privacy issues (Shahmoradi et al., 2017)? • Can we increase technology and information transfer by introducing appropriate intellectual property rights in the healthcare industry? (Fisman et al., 2004)?	Pathway 4

### Area of improvement 1: Anticipating the range of actor behaviours

As healthcare systems are becoming more open – for example, through the penetration of new market actors and the use of increasingly comprehensive and advanced health technologies – accurately determining the boundaries of an industry and its key actors is becoming more difficult. To model these systems, we must carefully model every interaction in them (Benbya et al., 2020), which first requires HIS researchers to identify potential actors in the ecosystem rather than predetermining assumed industry boundaries. As actors are not always evident, we follow Benbya et al. (2020) in proposing Salthe’s (1985) three-level specification, assisting researchers in identifying actors at the focal level of what is actually observed (e.g., hospitals, patients, and general practitioners) and its relations with the parts described at the lower level (e.g., administrators and legal professionals), taking into account entities or processes at a higher level in which actors at the focal level are embedded (e.g., national health system structures and supporting industries, such as the pharmaceutical or tech industries). These examples are only illustrative, and criteria for levels have to be suggested and discussed for each research endeavour.

To anticipate future developments in the healthcare industry, we also need to analyse the strategies and interests of actors for joining or staying in the healthcare industry. This is especially important because, like other complex socio-technical systems, the healthcare industry is made up of large numbers of actors that influence each other in nonlinear ways, continually adapting to internal or external tensions (Holland et al., 1996). If tension rises above a certain threshold, we might expect chaos or extreme outcomes. As these are not beneficial for the actors in the system, the eventual goal is to align actors’ interests and strategies across a specific range of behaviour to foster coevolution. This allows for multi-layered ecosystems that encourage joint business strategies in competitive landscapes, as well as the alignment of business processes and IT across actors (Lee et al., 2013).

### Area of improvement 2: Determining boundaries and fostering collaboration

Actors build the cornerstones of the healthcare industry. Thus, if we want to understand and capture its blurring boundaries, there is a need to understand the complex causality of interactions among heterogeneous actors. In particular, scholars have emphasized that, in complex systems, outcomes rarely have a single cause but rather result from the interdependence of multiple conditions, implying that there exist multiple pathways from an input to an output (Benbya et al., 2020). To capture interaction, we follow Kozlowski et al. (2013), who envisioned a positive feedback process including bottom-up dynamic interaction among lower-level actors (upward causation), which over time manifests at higher, collective levels, while higher-level actors influence interaction at lower levels (downward causation). As these kinds of causalities shape interaction within healthcare ecosystems as well as at their boundaries, HIS researchers need to account for multi-directional causality in the form of upward, downward, and circular causality (Benbya et al., 2020; Kim, 1992).

Understanding casualties among actors in the healthcare industry is important for harnessing the advantages of the blurring of boundaries – e.g., by making use of the emergent ecosystem for launching innovation cycles (Hacklin, 2008). However, first, HIS researchers increasingly need to consider the ecosystem perspective by investigating interactions among actors and the role of IS infrastructures in fostering collaborative health innovations. We propose a focus on radical innovation, which is necessary to address the diversity and interdependence of issues present in the healthcare industry by putting the patient at the core of all innovation efforts. HIS researchers, however, need to break down the boundaries between different innovation phases and innovation agencies, including a higher level of unpredictability and overlap in their time horizons (Nambisan et al., 2017). Notably, this requires actors in the healthcare industry to discover new meaning around advanced technologies and IS infrastructures whose design needs to facilitate shared meaning among a diverse set of actors, thereby fuelling radical digital innovations (Nambisan et al., 2017).

### Area of improvement 3: Creating sustainable knowledge ecosystems

We define knowledge dissemination and sharing as the ultimate means of connecting actors and aligning actions within common frameworks to shape an inclusive healthcare ecosystem. Paving the way for inclusive healthcare ecosystems is thus necessary to address the current shortcomings of HIS research as elaborated in the previous section.

Addressing knowledge dissemination and sharing is thereby of the utmost importance as we look at the healthcare industry in the current phase of emergence. This means that the industry might go through several transition phases in which existing actors, structures, and causal relationships dissipate and new ones emerge, resulting in a different set of causal relationships and eventually altering knowledge claims (Benbya et al., 2020). Creating a permeable and sustainable knowledge management system is necessary to ensure the transfer of knowledge for the best outcomes for the patient while securing the intellectual property rights and competitive advantages of diverse actors such as hospitals and other healthcare providers.

To be precise, we argue that to design sustainable knowledge management systems, HIS researchers need to implement systems with structures that create mutual benefits – i.e., encourage knowledge dissemination and sharing (e.g., open innovation) by actors in the healthcare industry. In a comprehensive and sustainable knowledge management system, however, not only corporations but also patients should be encouraged to share knowledge. Using this information, researchers and health service providers will be enabled to create optimized infrastructures, processes, and products (e.g., for predictive algorithms that improve treatment accuracy, or for assessing the likelihood of the occurrence of certain diseases and even of pandemics). At the same time, the trustworthiness of predictions and the anonymity of health information (and thus privacy) must be ensured. Bridging this duality of data sharing and knowledge dissemination, on the one hand, and protection of health information, on the other, is therefore essential for future HIS research.

## Conclusion

This paper analyses the HIS literature within the IS research domain, prompted by the question of whether IS researchers are prepared to capture and anticipate exogenous changes and the consequences of current developments in the healthcare industry. While this review is limited to insights into the IS research domain and does not claim to offer insights into the health literature in general or related publications (e.g., governmental publications), we disclose several shortcomings and three key issues. Based on these, we provide initial guidance on how IS research can develop so that it is prepared to capture the expected large and long-lasting changes from current and possible future pandemics as well as the necessary adaptation of global healthcare industries affecting human agencies and experiences in all dimensions. Thus, while adaptations in the healthcare industry are already emerging, IS researchers have yet to develop a more comprehensive view of the healthcare industry. For this purpose, we provide a research agenda that is structured in terms of three areas of improvement: anticipating the range of actors’ behaviours, determining boundaries and fostering collaborations among actors in the healthcare industry, and creating sustainable knowledge management systems. In particular, addressing these areas will assist IS researchers in balancing the shortcomings of current HIS research with the unique contribution that IS research plays in analysing, advancing, and managing the healthcare industry. We are confident that IS research is not only capable of anticipating changes and consequences but also of actively shaping the future of the healthcare industry by promoting sustainable healthcare ecosystems, cultivating structures of mutual benefit and cooperation between actors, and realigning IS research to face the imminent transformation of the healthcare industry. IS research cannot contribute directly to solving the current pandemic problems; however, it can contribute indirectly triggering timely adaptations of novel technologies in global health systems, and proposing new processes, business models, and systematic changes that will prepare health systems to cope with increasing digitalization and emerging players whose push into the market enabled by the exogenous effects triggered by the pandemic.

While we are confident that the proposed research agenda based on the analysis of HIS literature provides fruitful arrays for being prepared in anticipating the future role of IS research for the healthcare industry, our results need to be reflected in light of their shortcomings. First and foremost, we recognize that the selection of literature, which is limited to the IS research domain, excludes other contextual factors that are not primarily considered by IS researchers. Thus, we cannot assume completeness, providing instead a broad overview of current issues in HIS research. In addition, possible biases may have arisen due to the qualitative analysis approach used. By independently coding and discussing codes to the point of theoretical saturation, we are confident that we largely eliminated biases in the thematic analysis. However, data saturation could not be achieved. This means that further insights could have emerged through the addition of other database searches and journals with a broader scope. Additionally, the initial sorting of papers into single defined phases of DSR research restricted multiple assignments that could have led to different results. However, we consider sorting as a necessary step of abstraction, especially given the large number of papers analysed.

We deliberately considered IS research, for which we have developed an agenda for potential future research avenues. For each of those avenues, researchers should go deeper into the subject matter in order to examine the complexity of the paths shown and to include them in the analysis (e.g., through in-depth case studies). However, it is also clear from the issues identified that IS researchers cannot solve current challenges by working on the pathways alone. In fact, the issues identified in the research agenda are only the starting point for further research, which should address the proposed issues step by step and in cooperation with other research disciplines. The latter is likely to generate further and deeper-rooted problems, as well as, in turn, future paths for research. Nevertheless, we are confident that this paper provides an important first step in opening up HIS research to better understand current developments in the healthcare industry. Further, by following and enhancing the proposed research pathways, we believe that HIS research can contribute to and support changes already taking place in the healthcare industry.

## Supplementary Information

Below is the link to the electronic supplementary material.

Supplementary file1 (DOCX 195 KB)
