# Supplementary material for: Keeping pace with the healthcare transformation: a literature review and research agenda for a new decade of health information systems research
Source: Electron Mark. 2021 Jul 17;31(4):901–21. doi: 10.1007/s12525-021-00484-1 (PMC8285287; doi:10.1007/s12525-021-00484-1)
Supplement: Supplementary file 1 — Supplementary file1 (DOCX 195 KB) [file 12525_2021_484_MOESM1_ESM.docx]

**Appendix**

**Appendix 1. Number of HIS publications in journals and conference proceedings over 30 years**

**
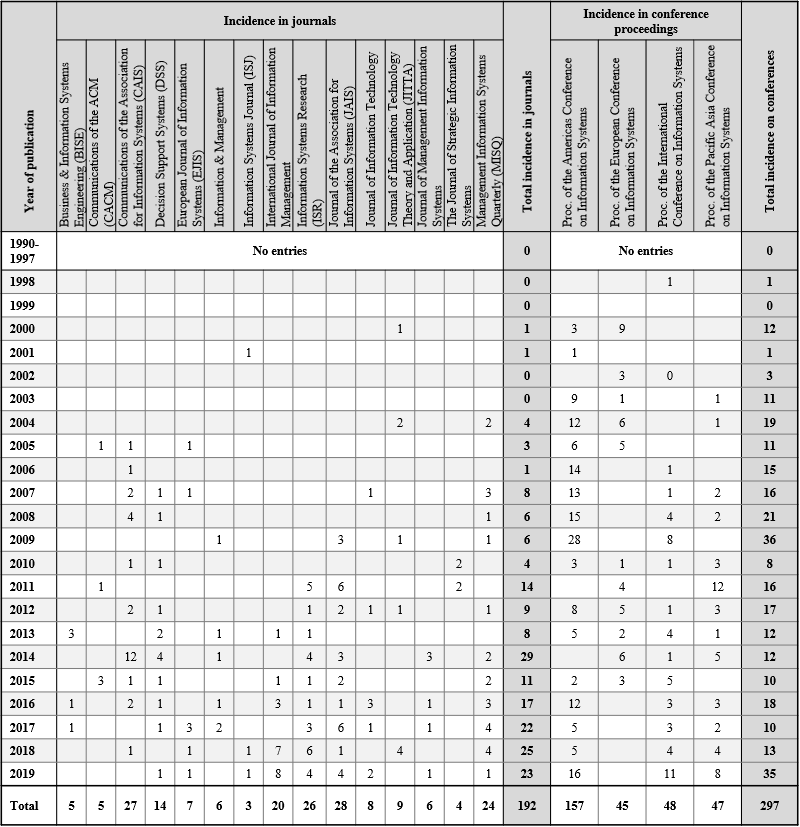
**

**Appendix 2. Number of HIS publications per theme label and phase**

**
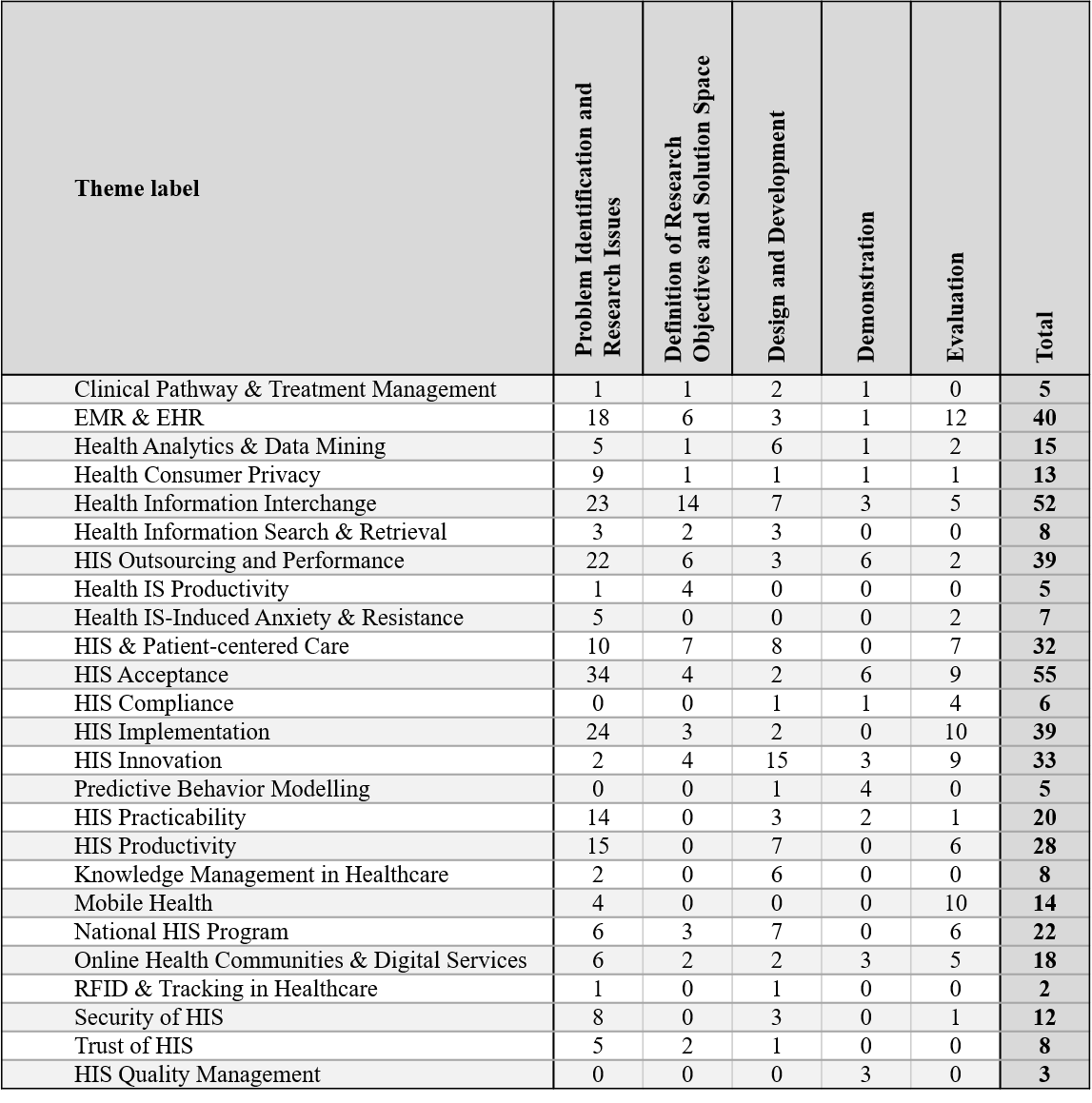
**
